# Bile acid patterns in commercially available oxgall powders used for the evaluation of the bile tolerance ability of potential probiotics

**DOI:** 10.1371/journal.pone.0192964

**Published:** 2018-03-01

**Authors:** Peng-Li Hu, Ya-Hong Yuan, Tian-Li Yue, Chun-Feng Guo

**Affiliations:** College of Food Science and Engineering, Northwest A&F University, Yangling, China; Maharshi Dayanand University, INDIA

## Abstract

This study aimed to analyze the bile acid patterns in commercially available oxgall powders used for evaluation of the bile tolerance ability of probiotic bacteria. Qxgall powders purchased from Sigma-Aldrich, Oxoid and BD Difco were dissolved in distilled water, and analyzed. Conjugated bile acids were profiled by ion-pair high-performance liquid chromatography (HPLC), free bile acids were detected as their *p*-bromophenacyl ester derivatives using reversed-phase HPLC after extraction with acetic ether, and total bile acids were analyzed by enzymatic-colorimetric assay. The results showed that 9 individual bile acids (i.e., taurocholic acid, glycocholic acid, taurodeoxycholic acid, glycodeoxycholic acid, taurochenodeoxycholic acid, glycochenodeoxycholic acid, cholic acid, chenodeoxycholic acid, deoxycholic acid) were present in each of the oxgall powders tested. The content of total bile acid among the three oxgall powders was similar; however, the relative contents of the individual bile acids among these oxgall powders were significantly different (*P* < 0.001). The oxgall powder from Sigma-Aldrich was closer to human bile in the ratios of glycine-conjugated bile acids to taurine-conjugated bile acids, dihydroxy bile acids to trihydroxy bile acids, and free bile acids to conjugated bile acids than the other powders were. It was concluded that the oxgall powder from Sigma-Aldrich should be used instead of those from Oxoid and BD Difco to evaluate the bile tolerance ability of probiotic bacteria as human bile model.

## Introduction

Bile acids are synthesized from cholesterol in liver hepatocytes in humans and most animals, stored in the gallbladder, secreted into the small intestine after ingestion of a fatty meal, efficiently reabsorbed by the distal small intestine and returned to the liver via the portal vein [1]. Bile acids are a digestive secretion that plays an important physiological role in the elimination of cholesterol from the body and in the intestinal solubilization and absorption of lipids [2].

Bile acid concentration ranges from ∼8% in the gallbladder to ∼0.2–2% in the intestine. These values are not absolute, though, as person-to-person variations in bile acid levels exist due to factors such as dietary intake [3]. In addition to their classical roles as detergents to aid in the process of digestion, bile acids are an important antimicrobial agent in the mammal gut [4]. Their antimicrobial mechanisms include inducing membrane damage, disturbing macromolecule stability [5], and dissipating the bacterial transmembrane electrical potential [6].

Probiotics are live microbial food supplements which, when administered in adequate amounts, exert various health benefits to the consumers [7]. These bacteria bring various health benefits to the host, such as modulation of immune responses, prevention of gastrointestinal infections, improvement of lactose metabolism, regulation of lipid metabolism, and antiobesity, anticancer, antiallergic, and antioxidative potentials [8, 9]. The current scientific consensus is that probiotics should be alive to exert their beneficial effect in the human gastrointestinal tract [10]. Moreover, to survive passage through the small intestine, probiotic strains must survive and grow in the presence of bile salts [11]. Hence, when evaluating the potential of using lactic acid bacteria as effective probiotics, it is necessary to evaluate their ability to resist bile [12, 13].

Due to the similarity between oxgall and human bile in bile acid composition, oxgall powder, a product derived from bovine bile, has been commonly used to assess the bile tolerance ability of potential probiotic strains at a concentration of 0.3% (wt/vol) instead of human bile [14–16]. In the international literature, the oxgall powders used for bacterial bile tolerance assay were mainly obtained from three manufacturers, namely Sigma-Aldrich (USA) [17, 18], Oxoid (UK) [19–21] and BD Difco (USA) [22, 23]. Little or no information has been reported on the total bile acid content and the content of the individual bile acids in commercially available oxgall powders. However, it is crucial to know this information in order to clarify which oxgall powder is closest to human bile.

This study aimed to analyze bile acid patterns in the three commercially available oxgall powders used for the evaluation of the bile tolerance ability of probiotic bacteria. Moreover, the results obtained were compared with those from human bile. This study provides a reference for the selection of commercially available products capable of simulating the bile environment of the human intestinal tract.

## Experimental material and methods

### Chemicals and reagents

All chemicals and solvents were of the highest purity commercially available. Standard bile acids (sodium salts), including glycochenodeoxycholic acid (GCDCA), glycodeoxycholic acid (GDCA), glycocholic acid (GCA), taurochenodeoxycholic acid (TCDCA), taurodeoxycholic acid (TDCA), taurocholic acid (TCA), chenodeoxycholic acid (CDCA), deoxycholic acid (DCA) and cholic acid (CA), were purchased from Sigma-Aldrich (St. Louis, MO, USA). Three different oxgall powder samples were obtained from Sigma-Aldrich (Product code B3883), Oxoid (Product code LP005, Basingstoke, Hampshire, UK) and BD Difco (Product code 212820, Sparks, MD, USA). Acetonitrile and methanol (HPLC grade) were supplied by Tedia (Fairfield, OH, USA). The derivatization reagents *p*-bromophenacylbromide and *N*, *N*-diisopropylethylamine, and the ion-pairing reagent tetrabutylammonium hydrogen sulfate were also purchased from Sigma-Aldrich.

### Analysis of conjugated bile acids

Samples of the three oxgall powders under study were dissolved in distilled water at a concentration of 0.3% (wt/vol), filtered through a 0.45-*μ*m nylon filter, and analyzed for conjugated bile acids using a Shimadzu LC-20A HPLC system (Kyoto, Japan) equipped with a TC-C18 reverse-phase column [5 *μ*m, 4.6 mm×250 mm; (Agilent Technologies, Santa Clara, CA, USA)]. The column temperature was maintained at 40°C and the flow rate was set at 1.0 mL/min. The sample injection volume was 20 *μ*L and ultraviolet detection was performed at 200 nm using a Shimadzu SPD-M20A photodiode array detector. Mobile phase solvent A was acetonitrile-water (60:40) containing 7.5 mM tetrabutylammonium hydrogen sulfate (pH 2.5), while solvent B was acetonitrile-water (30:70) containing 7.5 mM tetrabutylammonium hydrogen sulfate (pH 2.5) [24]. The mobile phase gradient increased linearly within 30 min from an initial concentration of 15% solvent A to a final concentration of 70% solvent A.

### Analysis of free bile acids

Samples of the three oxgall powders under study were dissolved in distilled water at a concentration of 1.0% (wt/vol) and filtered through a 0.45-*μ*m nylon filter. The oxgall solution (1 mL) was acidified with formic acid to pH 2.0 and then extracted three times with 3 mL of ethyl acetate. The combined organic extracts (4.0 mL) were evaporated to dryness under nitrogen stream at 40°C after dehydration with anhydrous sodium sulfate. The residue was dissolved in 2.5 mL of anhydrous acetonitrile–methanol (9:1) containing 5 g/L *p*-bromophenacylbromide. Subsequently, 25 *μ*L of *N*, *N*- diisopropylethylamine was added to catalyze the reaction as previously described [25], and incubated at 60°C for 30 min.

The free bile acid derivatives were analyzed using a Shimadzu LC-20A HPLC system equipped with a TC-C18 reverse-phase column (5 *μ*m, 4.6mm × 250 mm). The column temperature was maintained at 40°C and the flow rate was set at 1.0 mL/min. The sample injection volume was 20 *μ*L and ultraviolet detection was performed at 254 nm using a Shimadzu SPD-M20A photodiode array detector. Mobile phase solvent A was acetonitrile-water (70:30) at pH 3.10 adjusted with phosphoric acid, and mobile phase solvent B was acetonitrile. The mobile phase gradient increased linearly within 25 min from an initial concentration of 0% of the solvent B to a final concentration of 80% of the solvent B.

### Analysis of total bile acid

The total bile acid content was determined enzymatically by measuring the aqueous solution of the oxgall powders (0.3%, wt/vol) using a Hitachi 7180 automatic biochemical analyzer (Hitachi, Tokyo, Japan) coupled with a commercial kit from BioSino Biotechnology and Science Inc. (Beijing, China) as described previously [26].

### Statistical analysis

Data are expressed as mean ± standard deviation (SD). Statistical analysis was performed by one-way analysis of variance (ANVOA) followed by Tukey's multiple comparison tests using SPSS 18.0 software package (SPSS Inc., Chicago, IL, USA). A difference was considered statistically significant when *P* < 0.05.

## Results

The liquid chromatograms obtained from the analysis of the bile acid compositions of the three oxgall powders showed baseline separation and symmetrical sharp peaks for nearly all the conjugated bile acids (S1 Fig) and the free bile acids modified as *p*-bromophenacyl esters (S2 Fig) under the present chromatographic conditions. No severe peak tailing or leading was observed for any of the analytes. The major conjugated bile acids in the oxgall powders were identified as TCA, GCA, TDCA, GDCA, TCDCA and GCDCA, whereas the major free bile acids were characterized as CA, DCA and CDCA. The sum of the content of these individual bile acids in each of the oxgall powders exceeded 98% of total bile acids content determined with the enzymatic colorimetric method. The content of individual bile acids in the oxgall powders is summarized in Table 1. The content of the individual bile acids in each oxgall powder was significantly different (*P* < 0.05). In each oxgall powder, TCA was the most abundant conjugated bile acid, followed by GCA, TDCA, GDCA, TCDCA, and GCDCA, whereas CA was the most abundant free bile acid, followed by DCA and CDCA.

**Table 1:** Content of individual bile acids and total bile acid in oxgall powder from different manufactures.

Bile acid	Sigma-Aldrich		Oxoid		BD Difco	
	% (wt/wt)	mmol/kg	% (wt/wt)	mmol/kg	% (wt/wt)	mmol/kg
GCA	24.21 ± 0.06^a^	519.88 ± 1.36^A^	20.20 ± 0.03^c^	433.81 ± 0.64^C^	23.05 ± 0.11^b^	494.99 ± 2.31^B^
GDCA	5.05 ± 0.02^b^	112.34 ± 0.44^B^	4.39 ± 0.02^c^	97.70 ± 0.35^C^	5.55 ± 0.14^a^	118.67 ± 0.57^A^
GCDCA	1.38 ± 0.01^a^	30.66 ± 0.19^A^	1.19 ± 0.00^b^	26.53 ± 0.08^B^	1.39 ± 0.02^a^	30.85 ± 0.36^A^
TCA	30.53 ± 0.08^b^	592.00 ± 1.55^B^	31.64 ± 0.07^a^	613.65 ± 1.43^A^	30.00 ± 0.13^c^	581.85 ± 2.48^C^
TDCA	5.51 ± 0.05^c^	122.55 ± 1.16^B^	7.29 ± 0.07^a^	145.79 ± 1.45^A^	6.04 ± 0.04^b^	120.94 ± 0.81^B^
TCDCA	1.51 ± 0.01^c^	33.48 ± 0.27^B^	1.67 ± 0.02^a^	37.22 ± 0.35^A^	1.53 ± 0.01^b^	30.68 ± 0.25^C^
CA	0.58 ± 0.02^c^	13.58 ± 0.44^C^	0.95 ± 0.03^b^	21.98 ± 0.81^B^	2.87 ± 0.05^a^	66.67 ± 1.14^A^
DCA	0.09 ± 0.00^c^	2.09 ± 0.08^C^	0.11 ± 0.00^b^	2.77 ± 0.01^B^	0.50 ± 0.01^a^	12.15 ± 0.16^A^
CDCA	0.02 ± 0.00^c^	0.55 ± 0.05^C^	0.03 ± 0.00^b^	0.79 ± 0.03^B^	0.10 ± 0.00^a^	2.37 ± 0.03^A^
Total bile acid	—	1440.51 ± 20.83^AB^	—	1402.16 ± 15.15^B^	—	1460.66 ± 18.70^A^

Data are expressed as mean ± SD (*n* = 4). Means in the same column not sharing a common lowercase superscript letter are significantly different from each other (*P* < 0.05). Means in the same row not sharing a common uppercase superscript letter are significantly different from each other (*P* < 0.05).

Abbreviations: GCA, glycocholic acid; GDCA, glycodeoxycholic acid; GCDCA, glycochenodeoxycholic acid; TCA, taurocholic acid; TDCA, taurodeoxycholic acid; TCDCA, taurochenodeoxycholic acid; CA, cholic acid; DCA, deoxycholic acid; CDCA, chenodeoxycholic acid.

**Fig 1:**
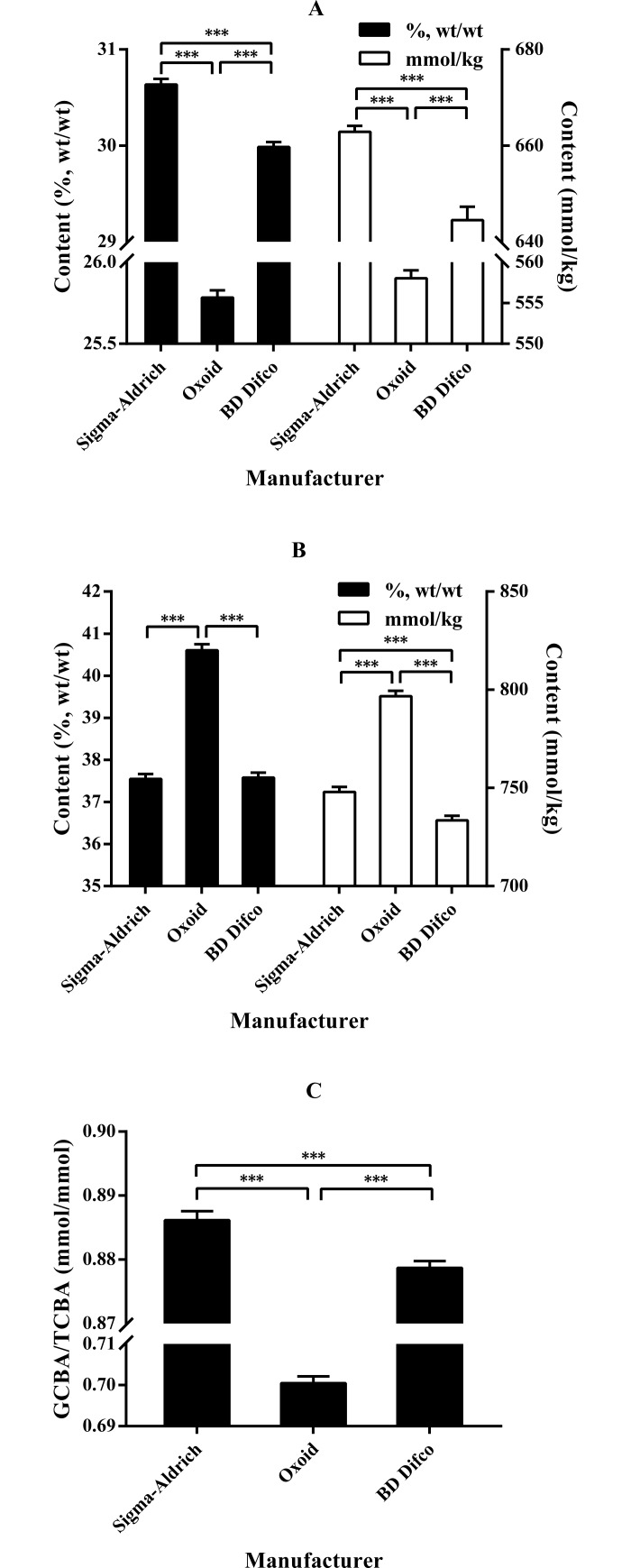
**Comparison of the contents of GCBA (A), TCBA (B), and GCBA/TCBA (C) in oxgall powders from the three manufacturers.** ***P < 0.001. Data are expressed as the mean ± SD (*n* = 4). Abbreviations: GCBA, glycine-conjugated bile acid; TCBA, taurine-conjugated bile acid.

Fig 1 shows the content of glycine-conjugated bile acids (GCBA) and taurine-conjugated bile acids (TCBA), and the GCBA to TCBA ratio in oxgall powders from different manufacturers. There were significant differences in these parameters among the oxgall powders (*P* < 0.001). The oxgall powder from Sigma-Aldrich showed the highest GCBA content, followed by those from BD Difco and Oxoid, whereas the oxgall powder from Oxoid showed the highest TCBA content, followed by those from Sigma-Aldrich and BD Difco. The oxgall powder from Sigma-Aldrich showed the highest GCBA to TCBA ratio, followed by those from BD Difco and Oxoid.

**Fig 2:**
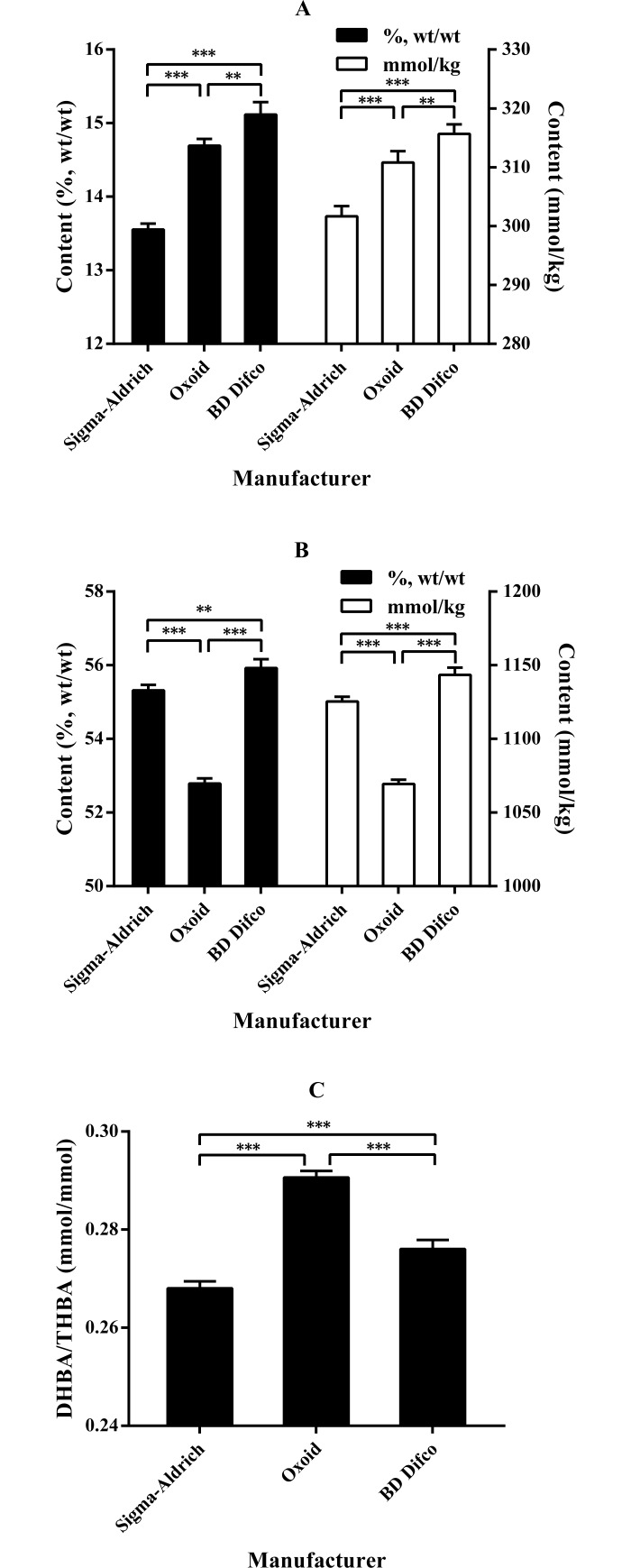
Comparison of the contents of DHBA (A), THBA (B) and DHBA/THBA (C) in oxgall powders from the three manufacturers. **P < 0.01 and ***P < 0.001. Data are expressed as the mean ± SD (*n* = 4). Abbreviations: DHBA, dihydroxy bile acids; THBA, trihydroxy bile acids.

Fig 2 shows the content of dihydroxy bile acids (DHBA) and trihydroxy bile acids (THBA) and the DHBA to THBA molar ratio in oxgall powders from different manufacturers. There were significant differences in these parameters among the oxgall powders (*P* < 0.01 or *P* < 0.001). The oxgall powder from BD Difco showed the highest DHBA and THBA content, whereas those from Sigma-Aldrich and Oxoid showed the lowest content of DHBA and THBA, respectively. The oxgall powder from Oxoid showed the highest DHBA to THBA molar ratio, followed by those from BD Difco and Sigma-Aldrich.

**Fig 3:**
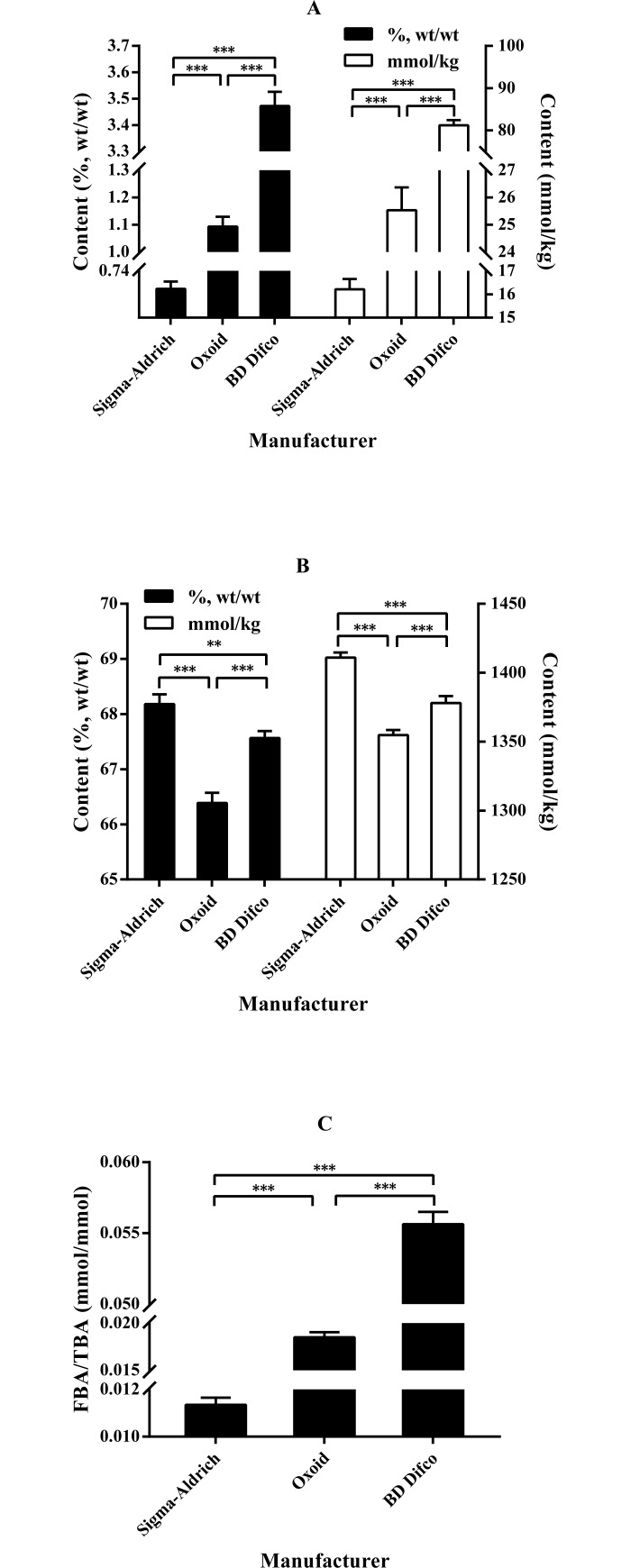
**Comparison of the content of FBA (A), CBA (B), and FBA/TBA (C) in oxgall powders from the three manufacturers.** **P < 0.01 and ***P < 0.001. Data are expressed as the mean ± SD (*n* = 4). Abbreviations: FBA, free bile acids; CBA, conjugated bile acids; TBA, total bile acids.

Fig 3 shows the content of free bile acids (FBA), conjugated bile acids (CBA), and the FBA/TBA ratio in oxgall powders from different manufacturers. There were significant differences in these parameters among the oxgall powders (*P* < 0.01, or *P* < 0.001). The oxgall powder from BD Difco showed the highest FBA content, followed by those from Oxoid and Sigma-Aldrich, whereas the oxgall powder from Sigma-Aldrich showed the highest CBA content, followed by those from BD Difco and Oxoid. The oxgall powder from BD Difco showed the highest FBA to TBA ratio, followed by those from Oxoid and Sigma-Aldrich.

## Discussion

Bile is a yellow-green aqueous solution whose major constituents include 70% bile salts, 22% phospholipids, 4% cholesterol, 3% proteins, and 0.3% bilirubin [27]. The core antimicrobial constituents in bile are conjugated bile acids formed from cholesterol in the liver cells. In the gallbladder, duodenum, and the jejunum, bile acids are present almost exclusively as glycine or taurine derivatives [28]. The human biliary bile acids consist mainly (~ 96%) of GCA, GCDCA, GDCA, TCA, TCDCA, and TDCA in a molar ratio of ~ 6:6:4:3:3:2 [29].

Each of the 9 individual bile acids could be detected in the three oxgall powders, and the majority of them were present in the form of conjugate either with glycine or taurine. Although these results are consistent with the findings on the major composition of human bile acids [29], the three oxgall powders differed from the human bile in the relative abundance of the individual bile acids. GCBA, DHBA, and FBA are more hydrophobic and, therefore, have faster transmembrane movement than TCBA, THBA, and CBA, respectively [30]. Consequently, GCBA, DHBA, and FBA have greater antibacterial activities than TCBA, THBA, and CBA, respectively [6, 31]. Accordingly, the higher GCBA to TCBA, DHBA to THBA, and FBA to CBA ratios, the greater the antimicrobial ability of bile will become when total bile acid concentration added to culture media is kept constant.

Because the oxgall powder from Sigma-Aldrich was the closest to human bile in the above parameters, it appears to be the best model for evaluating the bile tolerance ability of potentially probiotic strains. Considerable variation was also found in GCBA/TCBA, DHBA/THBA, and FBA/CBA among the three oxgall powders. One possible explanation for this discrepancy is that different bovine species or production technologies used by the manufacturers differ from each other. Moreover, the high FBA content in the oxgall powder from BD Difco might be due to chemical or microbial deconjugation of CBA.

Oxgall powders are also used as raw materials in the pharmaceutical industry to manufacture the therapeutic agents CDCA and ursodeoxycholic acid (UDCA) [32]. However, the oxgall powders analyzed in this study as biological agents have completely different bile acid compositions compared to those used as raw materials in the pharmaceutical industry. In fact, the latter oxgall powders usually undergo alkaline hydrolysis to release the precursor CA for the synthesis of CDCA and UDCA. Particularly, they mainly contain CA and DCA and small amounts of CDCA, and do not contain nearly any conjugated bile acids [33].

## Conclusions

Nine bile acids (i.e., TCA, GCA, TDCA, GDCA, TCDCA GCDCA, CA, CDCA, and DCA) were detected in all three oxgall powders from Sigma-Aldrich, Oxoid, and BD Difco. Although there was not considerable difference in the content of total bile acid among the three oxgall powders, significant difference was found in the relative content of the individual bile acids. Since the oxgall powder from Sigma-Aldrich was the most similar to human bile in the GCBA to TCBA, DHBA to THBA, and FBA to TBA ratios compared to those from Oxoid and BD Difco, it is the most suitable for evaluating the bile tolerance ability of probiotic bacteria instead of human bile.

## Supporting information

S1 Fig **Separation of conjugated bile acids in a standard mixture (0.8 mM each) (A) and aqueous solution of oxgall powder from Sigma-Aldrich (B), Oxoid (C), and BD Difco (D).** Peak identification: 1, GCA; 2, TCA; 3, GCDCA; 4, GDCA; 5, TCDCA; 6, TDCA. Abbreviations: GCA, glycocholic acid; TCA, taurocholic acid; GCDCA, glycochenodeoxycholic acid; GDCA, glycodeoxycholic acid; TCDCA, taurochenodeoxycholic acid; TDCA, taurodeoxycholic acid.
(EPS)

S2 Fig **Separation of *p*-bromophenacyl esters of free bile acids in a standard mixture (0.1 mM each) (A) and aqueous solution of oxgall powder from Sigma-Aldrich (B), Oxoid (C), and BD Difco (D).** Peak identification: 1, CA; 2, CDCA; 3, DCA. Abbreviations: CA, cholic acid; CDCA, chenodeoxycholic acid; DCA, deoxycholic acid.
(EPS)
